# Implementation of Integrated Service Networks under the Quebec Mental Health Reform: Facilitators and Barriers associated with Different Territorial Profiles

**DOI:** 10.5334/ijic.2482

**Published:** 2017-03-10

**Authors:** Marie-Josée Fleury, Guy Grenier, Catherine Vallée, Denise Aubé, Lambert Farand

**Affiliations:** 1 Department of Psychiatry, McGill University, Canada; 2 Douglas Mental Health University Institute Research Centre, 6875 LaSalle Blvd., Montreal, Quebec, Canada; 3 Rehabilitation Department, Laval University, Quebec, Canada; 4 Department of Social and Preventive Medicine, Laval University, National Public Health Institute of Québec, Canada; 5 Department of Health Administration, Policy and Evaluation, School of Public Health, University of Montreal, Montreal, Quebec, Canada

**Keywords:** Mental health reform, implementation, integrated service networks, territories, integration strategies

## Abstract

**Introduction:**

This study evaluates implementation of the Quebec Mental Health Reform (2005–2015), which promoted the development of integrated service networks, in 11 local service networks organized into four territorial groups according to socio-demographic characteristics and mental health services offered.

**Methods:**

Data were collected from documents concerning networks; structured questionnaires completed by 90 managers and by 16 respondent-psychiatrists; and semi-structured interviews with 102 network stakeholders. Factors associated with implementation and integration were organized according to: 1) reform characteristics; 2) implementation context; 3) organizational characteristics; and 4) integration strategies.

**Results:**

While local networks were in a process of development and expansion, none were fully integrated at the time of the study. Facilitators and barriers to implementation and integration were primarily associated with organizational characteristics. Integration was best achieved in larger networks including a general hospital with a psychiatric department, followed by networks with a psychiatric hospital. Formalized integration strategies such as service agreements, liaison officers, and joint training reduced some barriers to implementation in networks experiencing less favourable conditions.

**Conclusion:**

Strategies for the implementation of healthcare reform and integrated service networks should include sustained support and training in best-practices, adequate performance indicators and resources, formalized integration strategies to improve network coordination and suitable initiatives to promote staff retention.

Faced with rising demands for healthcare amid widespread economic uncertainty and budgetary restraint, health systems are obliged to undergo reform. Reform implementation represents a critical transitional period between the planning stages of system transformation and integration of reforms by service managers and providers. Research [1,2,3] suggests that nearly two thirds of reforms fail during this implementation period. Successful implementation involves specific activities affected by a multitude of factors ranging from characteristics of the reform itself, to organizational, environmental, and individual features. Innovation is easier to implement when core components are well-known and defined [4]; implementation fares better with simple, specific interventions rather than complex and lengthy interventions requiring major change at the organizational or practice levels [5,6]. Other factors at play include the political, economic, social and cultural context, as well as health system characteristics [1]. Important organizational-level issues include leadership [7]; financial and human resources [8,9]; staff retention [6,10]; receptivity to change [11,12]; and experience with inter-organizational collaboration [13]. A number of integration strategies at the administrative and clinical levels have been found to facilitate both reform implementation and organizational integration [14].

Mental health (MH) systems provide a rich arena for studying the implementation process in healthcare reform, as MH, and depression particularly, are expected to represent the primary cause of morbidity in developed countries by 2030 [15]. Most developed countries have engaged in MH system reform over the past two decades to improve system efficiency and better respond to client needs [13,16,17], focusing on increased accessibility, continuity and quality of services, and adopting innovations such as recovery-oriented practices. MH service delivery has shifted from hospital to community [18,19], reinforcing MH primary care and integrating primary with specialized MH services including substance use disorder (SUD) treatment [12,20]. There is also widespread interest in evidence-based practices such as cognitive behaviour therapy (CBT) and assertive community treatment (ACT) [21].

The promotion of integrated service networks is at the heart of MH reform in most countries, and certainly in the case of Québec. The aim of integrated services networks is to improve access, quality and continuity of care for clients with complex needs, including service users with severe MH disorders (MHDs) or co-occurring MHDs and SUDs [22]. Integration takes the administrative (or functional), clinical and professional dimensions into account, as well as relationships among various organizations [23]. Integration may be vertical, when all primary and specialized services are delivered by a single services provider, for example; or horizontal, as when links are created between primary care and specialized MH services [24]. Studies have found associations between the implementation of integrated services and positive outcomes in terms of decreased length of stay in hospitals, and fewer visits to emergency rooms [25,26], as well as better teamwork and job satisfaction among health professionals working in integrated teams [26,27].

While substantial literature describes barriers to implementing MH reforms at the national level [13,17,28], few studies have evaluated the degree of implementation at regional or local levels, or accounted for territorial diversity in terms of size, service delivery systems, healthcare practices, populations, etc. Yet successful implementation hinges on the degree to which innovations adapt to local needs [1,6]. Here Quebec provides an interesting case as one Canadian province that aimed to increase the integration and efficiency of 93 local service networks established in 2005 in the context of a major health and social service reform, within 15 health care regions, each with its own governing agency. The networks are highly diverse in terms of geography, population characteristics, and availability of MH resources.

Health care delivery in Canada relies mainly on a public system managed by the provincial governments, with financial support from the federal government. In Quebec, health and social services are integrated within a single overarching administration. As of April 2015, health care delivery was organized into nine service programs (e.g. MH, SUD, etc.), and managed at three regulatory levels: provincial, regional, and local. The Quebec Ministry of Health and Social Services assumes general governance and control over provincial healthcare. Regional health agencies are responsible for planning, organizing, coordinating, budgeting and evaluating healthcare and social services in their respective regions. As part of the 2005 health system reform, health and social service Centres (HSSC) for each of the local service networks were created from the merger of general hospitals, local community health centres and nursing homes. The HSSCs were responsible for service integration and quality care in health and social services for their respective local networks [29]. They were also mandated to develop strategic care planning for each healthcare service program, including MH, in conjunction with local service providers, with an overall aim of developing integrated MH service networks.

Within these networks, specialized MH services are offered by a general hospital psychiatric department or a psychiatric hospital; whereas primary care services for common MHDs (e.g. depression, anxiety) are provided by local community health centres. Community organizations provide other primary care services such as crisis centres and hot-line services, counselling, intensive case management, self-help groups, day centres, supported employment, and short or medium-term community-based as well as institutional housing. General practitioners and psychologists working in private clinics complete the Quebec mental health service system.

The aforementioned Quebec reform, or MH Action Plan 2005–2010 [30] also followed current healthcare trends aimed at promoting recovery among MH clients by focusing on primary care services that would foster community integration, service continuity and better service provider-client collaboration for people with MHDs living in the community; clients were transferred to specialized MH services as needed [30].

The main thrust of the MH Action Plan was to implement new structures and services. A one-stop service was established in networks with 50,000 or more inhabitants as the point of entry for accessing MH services, MH assessments were provided for clients referred by GPs, community organizations or inter-sectorial resources (e.g. SUD rehabilitation centres). The implementation of multidisciplinary MH primary care teams in the HSSCs was also endorsed. In order to sustain and enhance primary care, the MH Action Plan recommended shared-care initiatives whereby respondent-psychiatrists were hired to consult with, and support, HSSC-MH primary care teams and GPs in private clinics. The MH Action Plan also focused on the consolidation of treatment in HSSC-MH primary care teams for common MHDs, whereas other services aimed to promote recovery and community integration among clients with severe MHDs: intensive case management (ICM) and ACT, residential services including supervised housing, as well as crisis, suicide prevention, and employment integration services. Furthermore, the MH reform advanced strategies to facilitate collaboration between primary care and specialized MH services in the form of service agreements, liaison officers, and best-practices, e.g. strengths model and care pathways.

This study was original in evaluating a comprehensive MH reform that occurred in the context of the more global reform of the Quebec healthcare system described above. Similar to other international reforms, the Quebec MH reform was sparked by long wait times for psychiatric care, insufficient services for common MHDs, and an underperforming system insufficiently geared to quality care and client recovery. This study evaluated implementation of the Quebec MH Action Plan 2005–2015 in 11 local service networks organized into four territorial groups according to socio-demographic characteristics and MH services offered, and used a conceptual framework. We hypothesized that facilitators and barriers to implementation would be mainly associated with organizational characteristics.

## Methods

### Study Design and Data collection

The study employed mixed methods, triangulating different data sources across the 11 local MH service networks, which were identified in consultation with 20 MH decision makers and selected according to diversity of services offered, integration strategies, and uptake of best-practices [14,31]. Four profiles or groups emerged, based on key territorial characteristics: 1) presence of a psychiatric hospital (n = 3; “PH-Group”); 2) lack of any hospital with specialized MH services (n = 2; “WH-Group”); 3) less than 200 000 inhabitants, and availability of specialized MH services through a psychiatry department in a general hospital (n = 3; “SN-Group”); and 4) more than 200,000 inhabitants, and availability of specialized MH services through a psychiatry department in a general hospital (n = 3; “LN-Group”).

Data were collected from three sources: 1) documents concerning MH teams, organizations and networks, 2) structured questionnaires on primary and specialized MH services completed by managers and respondent-psychiatrists working in a shared-care model; and 3) semi-structured interviews with key network stakeholders involved in the reform. Quantitative data supplemented the qualitative data and vice versa (converging parallel triangulation method) [32]. A research advisory committee including eight Quebec decision-makers, and 11 designated respondents from each network helped with data collection and validated instruments.

Documentation obtained between November 2012 and March 2013 provided additional data on population and MH service characteristics, and on integration strategies, dynamics, and related challenges for each network. The self-administered questionnaires were completed between October 2013 and June 2014; they included standardized measures with categorical and continuous items and five- or six-point Likert scale responses. The MH services questionnaire for managers included items on: 1) client characteristics, 2) team profiles, 3) clinical activities, 4) network integration strategies, and 5) frequency and satisfaction of interactions involving network teams/organizations. Network integration strategies analysed were those usually reported on in the integration literature [14]. A question was asked in relation to each strategy (e.g. “In your opinion, to what extent, has this dimension been adopted within your organization?”). The respondent-psychiatrist questionnaire investigated questions around shared-care, including: 1) client characteristics, 2) respondent-psychiatrist activities, and 3) respondent-psychiatrist impact on MH services. The questionnaires took 120 minutes for managers, and 90 minutes for respondent-psychiatrists, to complete.

Interview guides for the qualitative research were developed and adapted to different stakeholder groups: regional managers; directors or managers of primary care teams or hospitals; respondent-psychiatrists; general practitioners (GPs); and community organization directors. Interviews were conducted between March and June 2014, and addressed issues related to: 1) MH client profiles; 2) implementation of the MH Action Plan; 3) MH network integration; and 4) facilitators and barriers to implementation of the Plan and to network integration. A single question was asked on each dimension (e.g. “In your opinion, what were the main factors that facilitated implementation of the reform in your network?”), followed by sub-questions eliciting additional information. The interviews and focus groups were audio-recorded, and transcribed; each participant was identified by number. All participants signed a consent form. The multi-site study protocol was approved by the Ethics Board of the Douglas Mental Health University Institute.

Individual interviews lasting 30–60 minutes were conducted in person or by telephone, and 60–90 minute focus groups conducted in person. Interviews were audio-taped and transcribed verbatim. Socio-demographic data were collected for all participants; anonymity and confidentiality were rigorously upheld.

## Data Analysis

**Figure 1:**
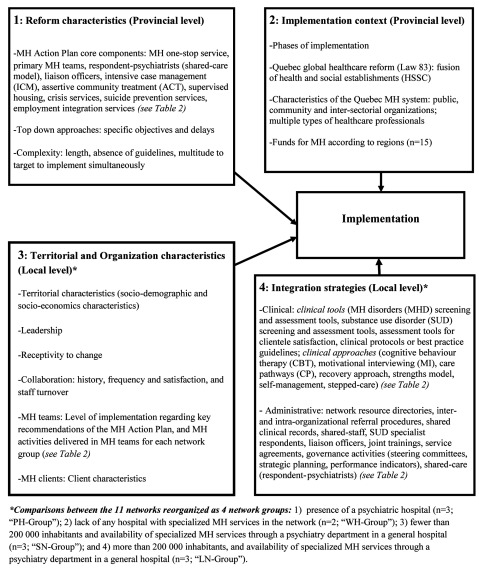
Conceptual framework.

A conceptual framework (Figure 1) based on existing implementation models [1,4,33,34] and related literature [6,10,11] guided the analysis. Implementation factors were organized into four main areas: two focused on provincial-level implementation: 1) reform characteristics and 2) implementation context; and two focused on each local level network group: 3) organizational characteristics, and 4) integration strategies.

Quantitative descriptive analysis using SPSS-17.0 software was used to compile data. Frequency distributions for categorical variables and mean values for continuous variables were computed. Qualitative analysis followed a six-step approach: 1) transcription of interviews; 2) preliminary readings; 3) selection and definition of classification units; 4) development of analytical framework (coding tree); 5) separation of content into units of meaning; and 6) data management with N-Vivo software, version 10 [35]. Coding was based on the interview topics listed above, allowing for inclusion of emerging issues, and further structured around mental health teams, organizations, and networks. Inter-rater reliability was verified for 20% of the codes. **Box 1** provides representative quotations from the qualitative interviews based on the conceptual framework (Figure 1).

## Results

### Description of the sample

In all, 97 MH service managers and 20 respondent-psychiatrists were recruited for the quantitative phase of the study; 90 managers and 16 respondent-psychiatrists participated, for a response rate of 91%. Most managers consulted their teams, and data banks, before completing the questionnaires. For the qualitative research, 110 key stakeholders were recruited, and 102 participated, for a response rate of 94%. In all, 78 qualitative interviews were conducted, 63 individual and 15 focus groups with a maximum of four participants each (Table 1).

**Table 1:** Socio-demographic Description of Professionals.

Variables	Categories	Questionnaires completed by managers/Coordinators of MH* specialized services (N = 48)	Questionnaires completed by managers/Coordinators of MH primary care teams (N = 33)	Questionnaires completed by managers/Coordinators of HSSC (N = 9)	Questionnaires completed by Respondent- psychiatrists (N = 16)	Interviews (N = 102)	Total: 208

**Average age [Mean (SD)]**		45.7	42.2	48.6	49.1	50.7	47.26
**Gender [n (%)]**	Female	30	25	5	6	69	135
	Male	18	7	4	10	33	72
**Current position [n (%)]**	Psychiatrists	0	–	0	16	7	23
	General practitioners (GPs)	0	–	0	–	10	10
	Psychosocial clinicians	9	7	0	–	4	20
	Regional managers	0		0	–	4	4
	Directors	0	–	3	–	35	38
	Program administrators/Coordinators	39	26	6	–	42	113
**Years of experience [Mean (SD)]**	In the current position	7.4	5.6	5.9	2.9	7.9	5.9
	In psychiatry	–	–	–	17.8	–	17.8
	In health and social services	–	–	–	–	23.1	23.1
	In mental health (MH)	–	–	–	–	19.4	19.4
	With adult populations (MH)	–	–	–	–	19.5	19.5
**Organizations**	Regional agencies	–	–	–	–	10	10
	Psychiatric hospitals (PHs)	23	–	–	4	14	41
	General hospitals (GHs)	25	–	–	3	9	37
	Health and social service centres		33	9	9	44	95
	Medical clinics		–	–	–	7	7
	Community organizations	–	–	–	–	18	18
**Types of territories [n (%)]**	With a PH	23	15	3	4	37	82
	Without specialized MH services	–	2	1	1	16	20
	> 200 000 inhabitants, with a psychiatric department in a GH	13	12	3	2	21	51
	< 200 000 inhabitants, with a psychiatric department in a GH	12	4	2	9	28	64

*MH: mental health.

### Reform characteristics – provincial level

The Quebec Ministry of Health and Social Services, through the MH Action Plan, supported standardized practices and a “top-down” approach. Staffing targets were projected for the consolidated primary care services, as follows: 20 psychosocial clinicians and 2 GPs per 100,000 inhabitants on HSSC-MH primary care teams; and respondent-psychiatrists at 3 hours per 50,000 inhabitants. Appropriate wait times were established for assessment at the MH one-stop service (n = 7 days), assessment at specialized MH services (n = 14 days), primary care treatment (n = 30 days) and treatment in specialized MH services (n = 60 days). However, implementation guidelines for the MH one-stop service and HSSC-MH primary care teams were not established in the MH Action Plan, resulting in uneven implementation of these structures across territories. Overall, the MH action Plan was very ambitious, involving a multitude of measures and targets that were difficult to implement simultaneously and leaving networks with some tough choices.

**Box 1. Representative Quotations**

**1) Reform characteristics (Provincial level)**

*Top-Down*: “You know they didn’t give us a choice, so we had to do some damage control” (45-psychiatrist, psychiatric hospital, PH-Group).

*Lack of a protocol on how to operationalize measures of the MH Plan*: “The implementation of the one-stop service was done at the beginning of the Mental Health (MH) plan but without any guidelines” (22-manager, Health and Social Service Centre (HSSC), PH-Group).

“Someone somewhere didn’t orient us well. That put the brakes on the development of primary care. Things had to be clear from the start; it’s like in intensive case management; the action plan talks about that but without defining it concretely” (39-manager, Regional Agency, SN-Group).

*Complexity*: « There is no end to the targets; there are so many… I would say that we need to prioritize our targets, because, if not, our staff will get exhausted from spreading themselves too thin” (54-manager, general hospital, LN-Group).

“It takes time to make changes. When the mental health action plan started off, we wanted to go too quickly, to implement everything at the same time. And in the end, that took much more time because we went too quickly at the beginning. Since then, we have let things go, and little by little we have initiated the changes that are going to be positive, that will continue” (02-manager, psychiatric hospital, PH-Group).

**2) Implementation context (Provincial level)**

*Merger*: “The links between primary care and specialized MH services are clear, and well defined. The lines are fluid because they are within the same institution” (68-manager, Regional Agency, LN-Group).

*General practitioners* (*GPs*) *interested in MH*: “They are very few, and are often overwhelmed with work. As well, GPs find that they are not sufficiently equipped to deal with this clientele” (21-manager, psychiatric hospital, PH-Group).

*Lack of resources*: “We have very limited financial resources. There are seven crisis centres in Montreal; yet, of the seven, we receive the least amount of financing for historical and political reasons, I would say. The need to make up for this shortfall interferes with our capacity to respond to client needs” (08-manager, community organization, PH-Group).

“We don’t yet have assertive community treatment (ACT) teams. We were also talking about services for co-occurring MH disorders (MHD) and substance use disorders (SUD)” (62-manager, HSSC, WH-Group).

“What we have noticed is that we are mainly lacking in residential resources where clients can live when they are in crisis situations, and this creates a lot of overflow in hospitals” (34-respondent-psychiatrist, SN-Group).

**3) Organizational characteristics (Local level)**

*Level of implementation of key recommendations of the MH Action Plan…*: “For clinicians, it was like a culture shock when we arrived here five years ago; and this is perhaps something we had not foreseen in terms of those who had already worked in the local community health centre (part of the HSSC) for a few years. Putting together clinicians who were transferred from hospital with young recruits doesn’t necessarily mean that a cohesive team is formed. So that was difficult; there was culture shock, and it was important to get beyond that stage” (07-manager, HSSC, PH-Group).

*Territorial characteristics*: “It isn’t easy sometimes when you have to visit a client, and the travel time takes longer than the time spent with him in therapy” (62-manager, HSSC, WH-Group).

*Leadership*: “We went from a period where specialized MH services played the entire leadership role, to a point where primary care services took over leadership. The director for MH primary care in the HSSC, who has been there for several years, is someone with the expertise, experience, and personality required of a leader” (16-manager, Regional Agency, PH-Group).

“I think that management at the Regional Agency became somewhat resentful from the time that the fusion with the HSSC was clinched, and that didn’t play well with their leadership role at the regional level” (55-manager, Regional Agency, WH-Group).

“The HSSC is the prime contractor, which however doesn’t impede the Regional Agency from playing an essential leadership role at the level of services, where it is necessary to promote best practices, etc.” (47-manager, Regional Agency, LN-Group).

*Receptivity to change*: “We undertook changes even before the arrival of the MH action plan” (70-manager, HSSC, LN-Group).

“Psychiatrists are the challenge. The head of the psychiatry department didn’t really believe in the MH Plan. For him, what was happening in primary care wasn’t good” (28-manager, Regional Agency, SN-Group).

*Clientele characteristics*: “There is a great deal of co-occurring MHD-SUD today. Taken separately, about 80% of the clientele has a SUD, while it’s 55–60% with a MHD. However, the two problems very much coincide, and these clienteles are very difficult to deal with” (43-manager, community organisation, SN-Group).

*Collaboration: history, frequency and satisfaction, and staff turnover*: “There was a big upheaval. What changed a lot were the personal contacts. They know people, but everyone is new; so there are no more cues” (62-manager, HSSC, WH-Group).

“I don’t want to generalize, but in some situations it’s as if the HSSC takes it for granted that they are the only ones with the competencies to intervene with clients dealing with MHD” (74-manager, Regional Agency, SN-Group).

“This is a region known for its collaboration before the changes – the transformations in 2007 and afterward. There was collaboration in place for years before the transformation” (47-manager, Regional Agency, LN-Group).

“There is a lot of staff turnover for various reasons: retirement, a desire to try other things, sick leaves, and maternity leaves among younger staff. Replacements may lack knowledge, experience, and training” (29-manager, HSSC, SN-Group).

“We are the crisis centre with the lowest salaries, and the least advantageous employee benefits; so this has an impact on staff retention” (08-manager, community organization, PH-Group).

**4) Integration strategies (Local level)**

*Clinical strategies: no verbatim* (*essentially quantitative investigation*).

*Administrative strategies*:

*Liaison officers*: “We give a lot of support to community organizations; there are liaison agents who go there; we give a lot of training, and we have created links with each organization” (12-manager, HSSC, PH-Group).

“We have what are called liaison nurses. There were at one time a lot of internal-external liaison agents but specifically for clients with serious or complex issues. They were able to do liaison with GPs, primary care providers, and bring everything together” (36-manager, HSSC, LN-Group).

*Joint training*: “A few years ago we put in place a joint training project with the SUD rehabilitation centre. We know that co-occurring MHD-SUD are increasingly present among our clients. At present we hold joint, in-house group sessions with workers from the SUD rehabilitation centre in the specialized MH services” (46-manager, HSSC, SN-Group).

“We don’t necessarily have what I would call a structured training program. There is training here and there, but not more than that” (50-GP, LN-Group).

*Mental Health National Centre of Excellence* (*MH-NCE*): “Henceforth we will have inclusion and exclusion criteria with regard to both intensive and variable case management (i.e. ACT and ICM). The MH-NCE was involved in developing this objective” (18-manager, psychiatric hospital, PH-Group).

“Thanks to the coaching from the MH-NCE, we have observed that service providers have made extraordinary gains in their understanding of intervention philosophy, their role, and the clientele that they should be targeting” (76-psychiatrist, SN-Group).

*Service agreements*: “What we try to work on a lot are the service agreements with our partners in order to offer them the support of a professional sponsor” (31-manager, HSSC, SN-Group).

“Community organizations had to give up some of their autonomy under the service agreements because we became a bit like sub-contractors” (58-manager, community organization, WH-Group).

*Governance activities*: “There are now some new steering committees that bring together directors from HSSCs or from general hospitals, but the community organizations are no longer invited. Personally, I think that we actually lost ground compared to the time when we occupied a bit more space on steering committees” (24-manager, community organization, PH-Group).

“The clinical project obliges us to sit together and discuss issues, with the result that our interventions now take the whole network more into account” (48-manager, HSSC, LN-Group).

*Performance indicators*: “In small territories, there isn’t a clear interface because everyone does a bit of everything” (61-manager, Regional Agency, WH-Group).

“There aren’t any qualitative indicators at present. I find this a bit of a gap because whatever we say about the extraordinary numbers at the emergency rooms, that doesn’t really describe practices in any depth. There is nothing to tell us, or make us reflect on, whether what we are doing is really good” (48-manager, HSSC, LN-Group).

*Shared-care*: “I think that primary care is not comfortable with psychiatry; recommendations are not always followed unfortunately” (34-respondent-psychiatrist, SN-Group.)

**Abbreviation list:**

ACT: Assertive community treatment

GP: General practitioner

HSSC: Health and Social Service Centre

ICM: Intensive case management

LN: Large network

MH: Mental health

MHD: Mental health disorder

MH-NCE: Mental Health National Centre of Excellence

PH: Psychiatric hospital

SN: Small network

SUD: Substance use disorder

WH: Without hospital

### Implementation context – provincial level

Although the MH Action Plan was introduced in 2005, most HSSC-MH primary care teams became operational as of 2008, and MH one-stop services by 2009; the first respondent-psychiatrists were appointed in 2010. Further delays occurred as implementation of the MH Action Plan coincided with the global reform of the Quebec health and social service system (Law 83) mentioned earlier [29]. Implementation of the MH Action Plan was facilitated where HSSC primary care and MH specialized services shared the same space, enhancing collaboration and continuity. By contrast, where HSSCs grew out of organizations with different missions, practices and cultures, staff and management turnover resulted and implementation stalled.

The MH Action Plan also reflected particular characteristics of the Quebec MH system, as a predominantly public system including an extensive community sector (e.g. crisis centres, self-help groups), private services (e.g. psychologists in private practice, residential services), and inter-sectoral resources (e.g. municipalities, school boards). Most of the MH budget had traditionally been allocated to inpatient services. Priority services (e.g. crisis services, housing, etc.) were only partially developed or consolidated, mainly due to inadequate funding. Community organizations, while recognized as key players in MH service delivery, were also underfunded. Moreover, significant regional disparities existed in relation to funding levels, while resource allocation tended to favour services for severe MHDs and specialized hospital services over primary care services for common MHDs. No additional funds were forthcoming to support GPs working with MH clients in medical clinics.

The tendency of psychiatrists to settle in metropolitan areas like Montreal, or the national capital region (Quebec City), where they staffed psychiatric clinics or general hospitals, created further issues. Psychiatrists represented various cultures and schools of thought, some supporting a hospital-centred model and others a community model. Those with negative experiences around patient transfers to the community, a central aim of MH reform, had little confidence in primary care services. The mandatory respondent-psychiatrist position was only ratified in 2009 after a long and protracted negotiation between the Ministry and the Quebec Psychiatric Association. For their part, most GPs were comfortable treating common MHDs, yet reluctant to take on clients with severe or complex cases such as co-occurring MHD-SUD. Finally, approximately 80% of psychologists worked in private practice [36], and were not remunerated through the Quebec Medicare system, which precluded access to psychotherapy for most individuals without private insurance.

### Organizational characteristics – Comparisons among the four network groups

#### Territorial characteristics

Important territorial disparities affected the implementation of integrated service networks. PH-Group territories were characterized by very pronounced socio-economic inequalities, but were rich in MH resources. By contrast, the WH-Group comprised rural or semi-urban territories, with higher than average incidences of low-income individuals, but no specialized MH services and correspondingly low funding for MH. The SN-Group included remote territories widely affected by poverty, also with below-average MH funding. The LN-Group included urban or semi-urban territories with higher than average individual income levels, but below-average funding for MH. For the WH-SN-Groups, lack of public transportation further hindered access to services for impoverished individuals. Transportation time needed in large WH-LN territories encroached on time available for treatment among clinicians. Within the WH-Group, non-francophone minorities faced linguistic barriers in accessing services.

#### Leadership

Under the MH Action Plan, HSSCs had to assume network leadership, while relying on the Regional Agencies for funding, training and development of best practices. HSSCs in the LN-group reportedly assumed this role to perfection. However, the Regional Agency retained greater influence in the SN-Group, while its counterpart in the WH-Group gradually reduced involvement in MH as the HSSC assumed leadership. HSSCs in the PH-Group faced other challenges in assuming a leadership role, as most services in those territories had been under PH control historically. Overall, the HSSCs seemed well suited to a leadership role, whereas the quality of leadership offered by Regional Agencies tended to vary.

#### Receptivity to change

Most territories in the PH- and LN-Groups had transferred stabilized clients with severe MHD and their clinicians several years before introduction of the MH reform. As such, the reform served to formalize and facilitate this effort, while promoting service development for clients with common MHDs. However, reform was hampered in most territories by GPs who resisted using the MH one-stop service. Considerable information was disseminated before GPs finally began to appreciate this new structure, and to accept their loss of direct contact with psychiatrists. The MH reform also unsettled some psychiatrists, particularly those in the WH- and SN-Groups who were reluctant to change their practices. Some, in fact, refused all involvement in the reorganization of MH services, which created another barrier to change.

#### Collaboration: history, frequency and satisfaction, and staff turnover

Partnerships between public institutions and community organizations in the LN-group existed long before inception of the MH reform. Overall, the LN- and SN-Groups counted frequent and satisfactory interactions with organizations and services in their respective networks. By contrast, the PH-Group included large MH organizations that tended to function in silo; the reform enforced more satisfactory interactions among the partners. The WH-Group represented the fewest and least satisfying relationships among organizations and services (Table 4). Network collaboration in the PH- SN-Groups was primarily affected by high staff turnover due to the transfer of clinicians from hospitals to the HSSC-primary care teams. Staff retention was also an issue in MH community organizations where salaries were lower, and benefits less advantageous as compared with staff in public institutions.

#### Level of implementation concerning key recommendations of the MH Action Plan and MH activities delivered in MH teams

The prescribed ratio of multidisciplinary clinicians per 100,000 inhabitants in HSSC-MH primary care teams was best achieved in the WH- and SN-Groups; yet primary care teams were often implemented at the expense of specialized MH services. Teams in the PH-Group included many long-standing staff members transferred from PHs, who faced considerable challenges in adapting to work in primary care. The targets for GPs in primary care teams were not achieved by any territory in the PH- or SN-Groups; whereas SUD specialists were most abundant within the PH-Group, and psychiatrists in the LN-Group. The MH one-stop service was totally implemented in the PH- and LN-Groups. Recommended delays for evaluation and treatment in primary care were better achieved in the LN-Group. As for specialized services, the recommended ratios of professionals for ICM teams were not reached in the PH-Group, where ICM was offered mainly by community MH organizations. Projections for the respondent-psychiatrists were fully realized in the PH- and SN-Groups. Recommended delays for evaluation or treatment by specialized MH services were only attained in certain territories of the LN-Group (Table 2). In terms of other MH network activities, intensity of patient care was higher in the LN-Group for primary care and in the PH-Group for specialized services. Duration of client follow-up was higher in both primary and specialized care for the SN-Group (Table 3).

**Table 2:** Synthesis of the Mental Health (MH) Reform – implementation targets.

1-Quebec MH Reform: Targets achieved		PH-Group (n = 3)	WH-Group (n = 2)	SN-Group (n = 3)	LN-Group (n = 3)

***MH one-stop service***					
MH one-stop service in all networks with a population of 50,000 inhabitants or more		3 (100%)	1 (50%)	1 (33%)	3 (100%)
***Health and Social Service Centres (HSSC)-MH primary care teams (for adults)***					
20 multi-disciplinary MH clinicians/100,000		1 (33%)	1 (50%)	2 (67%)	1 (33%)
2 general practitioners (GPs)/100,000		0 (0%)	1 (50%)	0 (0%)	1 (33%)
Access to evaluation: 7 days		1 (33%)	1 (50%)	1 (33%)	2 (67%)
Access to treatment: 30 days		0 (0%)	1 (50%)	0 (0%)	2 (67%)
***Intensive case management (ICM)***					
ICM in HSSC		1 (33.3%)	2 (100%)	2 (67%)	2 (67%)
ICM offered by MH community organizations (but under the responsibility of the HSSC)		3 (100%)	0 (0%)	0 (0%)	3 (100%)
***Respondent-psychiatrists (shared-care model)***					
1 respondent-psychiatrist/50,000 (3 hours/service: to HSSC-MH Primary care teams and GPs)		3 (100%)	1 (50%)	3 (100%)	2 (67%)
***Specialized MH services***					
Access to evaluation in specialized MH services: 14 days		0 (0%)	N.A.	0 (0%)	2 (67%)
Access to treatment in specialized MS services: 2 months		0 (0%)	N.A	0 (0%)	2 (67%)
Assertive community treatment programs (ACT)		1 (33%)	N.A	1 (33%)	1 (33%)
**2- Main strategies to consolidate primary care or network integration, based on the literature [58]**					
***2.1 Clinical Strategies***					
**Evaluation/clinical tools:** Establish clinical standardization and rationalization to promote best practices.[14]	Screening tools for MHDs Screening tools for SUDs Assessment tools for MHDs Assessment tools for SUDs Assessment tools for client satisfaction Clinical protocols or best practice guidelines	Mainly implemented in the PH Group			
**Clinical Approaches (Best practices)**	**Cognitive behaviour therapy (CBT):** Psychotherapy aiming to change thinking and behaviour. Effective for most MHDs, including SUDs [59].		Mainly used in the WH-Group		
	**Motivational interviewing (MI):** Brief intervention aiming to engage motivation to change behaviour. Mainly effective for SUDs [60].		Mainly used in the WH-Group		
	**Care pathways:** Systematic interventions planned for integrating care between different organizational units, or between providers, for a well-defined group of clients and treatment periods. Originally established in physical health for acute care, for which it has been proven effective, this care process aims at enhancing continuity of care and system efficiency. It is applied currently in MH [61].		Mainly used in the LN-Group		
	**Recovery approach:** Personal journey that involves developing hope, a secure base and sense of self, supportive relationships, empowerment, social inclusion, coping skills, and meaning. In most longitudinal studies, recovery rates were 80% for bipolar disorders, 65% to 80% for major depression, 70% for SUDs and 60% for schizophrenia [62,63].		Mainly used in the SN-Group		
	**Strengths model:** Intervention focusing on the strengths and interests of the user rather than pathology and oriented toward achieving goals set by the user him/herself. Mainly effective for severe MHD [64].		Mainly used in the SN-Group		
	**Illness self-management:** Systematic provision of education and supportive interventions in order to increase skills and confidence of the client in managing his/her health problems. Mainly effective for depression [65].		Mainly used in the WH-Group		
	**Stepped-care:** Care delivery model in which interventions are performed hierarchically based on the intensity of client problems. Mainly effective for depression [66].		Little used, but more in the PH-Group		
***2.2- Administrative Strategies***					
**Referral mechanisms:** Network resource directories Referral procedure within organization Referral procedure between organizations Shared clinical records			Mainly implemented in the PH- and WH-Groups		
**Shared staff:** Professionals offering services across more than one organization to insure coverage of the required range of services and to intensify inter-organizational collaboration [14].			Little implemented, but more in the PH-Group		
**SUD specialist respondents:** Specialists in SUDs who hold case discussions with MH and other teams concerning SUDs, aiming to reinforce SUD expertise and interventions including SUDs and co-occurring MHD-SUDs.			Mainly implemented in the WH-LN-Groups		
**Liaison officers:** Professionals designated by an organization to relay information between departments of a single organization or between organizations serving the same clientele. [14].			Mainly implemented in the PH- and SN-Groups		
**Joint training:** A strategy to enhance collaborative environments by simultaneously training clinicians with different areas of expertise and/or from different services or organizations in a network [53].			Mainly implemented in the PH-Group		
**Service agreements:** Administrative strategy used for formalizing mechanisms to facilitate access and continuity of services between at least two organizations or programs in the same organization [14].			Mainly implemented in the LN-Group		

**Table 3:** Compositions and activities of mental health (MH) services.

Variables	Categories	PH-Group^a^		WH-Group^b^	SN-Group^c^		LN-Group^d^	
		
		Primary care (n = 15)	Specialized care (n = 23)	Primary care (n = 2)	Primary care (n = 12)	Specialized care (n = 13)	Primary care (n = 4)	Specialized care (n = 12)
		
		Mean %	Mean %	Mean %	Mean %	Mean %	Mean %	Mean %

**Composition of professional teams [n(Mean)]**	Psychologists	3.4	0.9	10.8	0.9	0.7	6.8	1.0
	Social workers	3.8	1.9	4.0	1.3	1.1	8.3	2.0
	Psycho-educators	3.2	2.0	10.0	0.9	1.8	7.0	0.9
	Nurses	2.4	3.9	7.3	1.2	5.8	3.0	9.6
	Psychiatrists	0.1	2.3	1.4	0.0	3.6	4.0	5.2
	General practitioners (GPs)	0.3	0.4	1.2	0.9	1.1	0.3	0.5
	Professionals in substance use disorders (SUD)	1.5	1.7	0.0	0.2	0.3	0.3	0.9
**Time allocated by teams to [n (%)]**	Treatment or intervention	53.0	61.8	67.5	49.4	71.1	56.6	63.2
	Evaluation	27.5	29.4	9.0	23.6	35.8	31.8	24.5
	Coordination with other teams	21.8	22.0	16.0	14.9	19.2	17.5	8.3
**Clientele followed-up [n (%)]**	Stabilized disorders	58.3		22.5	41.6		52.5	
	Common MH disorders (MHD)	44.3		30.0	16.5		33.8	
	Severe MHD	37.1		40.0	62.2		23.8	
	Personality disorders	41.5	29.0	20.0	14.4	33.8	12.5	24.8
	Chronic physical disorders	32.3		17.5	21.8		8.3	
	Co-occurring MHD-SUDs	37.5	47.1	50.0	31.2	50.1	32.3	33.3
	Suicidal ideations	27.8	33.2	45.0	14.8		33.8	28.8
	Co-occurring MHD and chronic physical disorders	25.5	36.3	25.0	27.0	43.6	15.8	22.9
	Problems with the law	5.5	22.1	20.0	11.7		4.0	15.7
	High users	21.1	34.6	22.5	14.1		4.5	11.2
	Psychotic disorders		50.2			48.2		51.4
	Mood disorders		40.9			21.0		35.8
	Anxiety disorders		26.7			24.3		26.4
	Bipolar disorders		27.9			18.7		20.4
**Frequency of visits [n (%)]**	Once or more/month	89.9	93.7	93.4	88.4	76.7	97.4	81.4
	Once/3 months	6.8	4.8	5.0	0.7	12.5	1.7	9.3
	Once/6 months	1.9	1.2	1.7	2.8	6.3	0.9	4.6
	Once/year	1.4	0.3	0.0	8.1	4.5	0.0	4.8
**Duration of client follow-up [n (%)]**	>1 year (%)	50	87.3	22.5	72.4	88.2	60.0	65.6
	< a year (%)	20.9	11.8	35.0	13.8	10.6	26.7	8.5
	< 6 months (%)	12.7	14.5	27.5	12.1	18.9	18.3	0.7
	< 3 months (%)	31.9	77.9	17.5	23.3	47.1	45.0	69.5
**Proportion of clientele referred to [n (%)]**	MH Community organizations	36.8	29.8	25.0	46.3	21.5	36.3	38.8
	Specialized MH services	16.2	34.8	25.0	23.1	25.7	7.5	13.0
	Intersectoral resources	5.1	14.6	22.5	15.9	3.9	11.7	10.0
	SUD rehabilitation centres	10.9	12.8	7.5	10.2	11.6	11.7	23.4
	Community organizations not in MH	13.9		20.0	21.8		7.5	
	HSSC^e^-MH Primary care teams		27.6			23.0		22.5

a: With a psychiatric hospital (PH); b: Without specialized MH services in the network; c: <200 000 inhabitants with psychiatric department in a general hospital (GH); d: >200 000 inhabitants with psychiatric department in a GH; e: Health and Social Services Centres (HSSC)-MH Primary care teams.

**Table 4:** Frequency of interactions with other services and organizations and satisfaction.

Variables	Categories	PH-Group^a^	WH-Group^b^	SN-Group^c^	LN-Group^d^

		Mean^d^	Mean^d^	Mean^d^	Mean^d^

**Frequency of interactions from HSSC^e^-MH^f^ primary care teams**	General practitioners (GPs) in medical clinics	3.4	3.5	2.7	3.5
	HSSC one-stop service	2.9	4.5	3.6	3.3
	HSSC general services	2.5	3.0	3.7	3.5
	Respondent-psychiatrists	4.7	3.8	4.6	4.4
	Emergency rooms	3.1	2.5	4.3	3.3
	Hospitalization units	3.1	2.8	3.9	3.4
	Day hospitals	3.0	2.3	3.3	2.9
	Community organizations	2.6	3.0	2.8	2.5
	Crisis centres	3.1	4.0	2.9	4.8
	SUD^g^ rehabilitation centres	3.5	4.0	2.8	3.3
**Satisfaction of interactions from HSSC-MH primary care teams**	GPs in medical clinics	3.4	3.0	4.1	3.5
	HSSC one-stop service	4.1	4.0	4.3	4.5
	HSSC general services	4.1	5.0	3.9	2.5
	Respondent-psychiatrists	4.6	3.5	4.4	5.0
	Emergency rooms	3.5	3.0	4.5	3.6
	Hospitalization units	3.9	2.7	4.2	3.5
	Day hospitals	4.4	3.5	4.8	4.8
	Community organizations	3.4	3.5	3.8	2.8
	Crisis centres	3.7	5.0	4.6	5.0
	SUD rehabilitation centres	4.9	4.0	4.3	4.0

a: PH: with a psychiatric hospital; b: WH: without an hospital in the network; c: SN: small networks (<200 000 inhabitants with psychiatric department in a general hospital); d: LN = large networks (>200 000 inhabitants with psychiatric department in a general hospital); d: Mean from 0 to 5; 5 = better; e: HSSC: Health and Social Services Centres; f: Mental health; g: SUD: Substance use disorders.

#### Client characteristics

While HSSC-MH primary care teams were mandated to serve common MHDs versus severe MHDs, this only occurred in the PH (44% vs 37%) and LN (34% vs 24%) groups (Table 3). Most clients (62%) followed in primary care for the SN-Group had severe MHDs. Deinstitutionalization had created a concentration of former inpatients from general hospitals living in communities within the SN-Group; the needs of these clients were not well supported, and their presence hindered access to primary care for clients with common MHDs. The WH-Group included clients with co-occurring MHD-SUD (40%), high suicide risk (45%) and/or legal problems (20%). Due to lack of resources, wait times for treatment were often long. Overall, clients with more complex profiles, e.g. multiple MHDs and/or co-occurring SUDs, physical or intellectual disorders, tended to receive treatment in specialized care. Personality disorders were also on the rise in all the networks, increasing frequency and time allocations for treatment.

***Strategies developed by HSSC-MH primary care teams for integration with specialized care services – Comparisons among the four network groups***

The HCCS-MH primary care teams introduced several consolidation strategies with modest overall results (Table 5). These strategies mainly aimed at supporting horizontal integration, i.e. links between primary care and specialized services [24,37]; they included clinical and administrative (functional) integration strategies. Clinical integration strategies included screening and needs evaluation tools and approaches, while administrative (functional) integration strategies included service referral procedures, liaison officers, service agreements and other processes facilitating inter-organizational collaboration.

**Table 5:** Integration strategies developed by HSSC^a^-MH primary care teams to consolidate care in their services or to integrated their services with specialized care.

Variables	Categories	PH-Group^b^	WH-Group^c^	SN-Group^d^	LN-Group^e^

Clinical Strategies		Mean^f^	Mean^f^	Mean^f^	Mean^f^

**Evaluation/clinical tools**	Screening tools for MHDs^g^	3.6	1.5	2.3	1.7
	Screening tools for SUD^h^	4.0	4.5	3.7	4.0
	Assessment tools for MHDs	3.6	2.0	2.7	2.7
	Assessment tools for SUDs	4.1	3.0	3.1	3.7
	Assessment tools for client satisfaction	2.5	2.0	1.9	2.7
	Clinical protocols or best-practice guidelines	2.9	5.0	3.1	3.7
	Cognitive behaviour therapy (CBT)	3.0	4.0	3.7	3.3
**Clinical Approaches**	Motivational interviewing (MI)	3.1	4.0	3.3	3.0
	Care pathway	3.0	3.0	2.3	3.7
	Recovery approach	2.7	2.5	3.4	2.7
	Strengths model	2.9	2.5	3.1	3.0
	Illness self-management	2.5	3.0	2.4	2.6
	Stepped care	2.5	1.0	1.4	2.3

**Administrative Strategies**		**Mean^f^**	**Mean^f^**	**Mean^f^**	**Mean^f^**

Network resource directories		4.9	4.0	4.0	4.0
Referral procedures within the organization		4.4	5.0	4.3	3.7
Referral procedures between organizations		4.3	5.0	3.9	4.0
Shared clinical records		4.2	4.5	3.7	2.3
Shared staff		2.3	2.0	2.1	1.3
Liaison officers		3.2	2.0	3.0	2.3
Joint training		3.4	2.0	2.7	2.7
Service agreements		3.0	2.5	3.1	3.7
SUD specialist respondents		2.2	3.0	2.4	3.0

a: HSSC: Health and Social Services Centres; b: PH: with a psychiatric hospital; c: WH: without an hospital in the network; d: SN: small networks (<200 000 inhabitants with psychiatric department in a general hospital); e: LN: large networks (>200 000 inhabitants with psychiatric department in a general hospital); f: Mean: from 0 to 5; 5 = greatest utilization; g: MHDs: Mental health disorders; h: SUDs: Substance use disorders.

Concerning the implementation of clinical integration strategies, the PH- and WH-Groups achieved the greatest success, particularly the PH-Group which implemented more standardized evaluation/clinical tools than other groups. SUD screening and assessment were the most frequently utilized clinical tools in all networks, whereas tools for assessing client satisfaction were rarely utilized. MH screening and assessment tools were used predominantly in the PH-Group. Clinical protocols or best-practice guidelines were fully implemented in the WH-Group, but only moderately in other groups.

Standardized clinical approaches for MHD treatment were under-developed in all groups, as opposed to evaluation/clinical tools. CBT was the main approach used everywhere except by the WH-Group. Motivational interviewing (MI) and care pathways (CP) were mainly adopted by the PH-Group; MI was also adopted by the WH-Group and CP by the LN-Group. The Recovery approach was especially popular in SN-Group networks.

Standardized administrative integration strategies were more widely implemented than evaluation/clinical tools and clinical approaches in particular; they included network resource directories, referral procedures within or between organizations, and shared clinical records. The PH-Group favoured use of network directories, whereas the WH-Group opted for other approaches. While respondents identified staff sharing in network organizations as a key strategy, this was not widely implemented. Integration of SUD specialist respondents into the HSSCs, liaison officers and joint training were also targeted, but not well implemented. Liaison officers in the form of nurses attached to emergency room were widely implemented in the PH- and SN-Groups; nurses assessed and referred individuals with SUDs to treatment. Other liaison agents strengthened links between primary care and specialized services, and between HSSCs and partners, including GPs and community organizations.

Joint training was implemented mainly in the PH-Group, with the accent on evaluation and follow-up of clients at high risk for suicide. Motivational interviewing for personality disorders, SUDs, and co-occurring MHD-SUDs was another topic of interest. Training on ICM and ACT teams was conducted especially in the PH- and SN-Groups, and in the LN-Group to some extent. Aside from joint training offered to ACT and ICM teams, training provided to GPs and clinicians in MH services stemmed from local initiatives or clinician requests. The MH National Centre of Excellence (MH-NCE), an agency created under the Quebec Ministry of Health and Social Services to promote the reform, conducted training on ACT and ICM teams, and was viewed by SN-Group respondents as helpful in operationalizing MH one-stop services and introducing best practices in primary care.

Service agreements were reportedly well implemented in all networks, mainly in the LN-Group. Agreements were generally signed between the HSSCs and inter-sectorial resources in order to better coordinate services on specific issues or with SUD rehabilitation centres for shared treatment of clients with co-occurring MHD-SUDs. While some MH community organizations also signed service agreements, others refused. In the WH-Group, service agreements jeopardized the autonomy of community organizations. A cultural change was required within the PH-Group before their agreements could be formalized; that is, PHs had to recognize that they were not the sole purveyors of MH services.

Governance and shared-care were the last two strategies developed in the context of the Quebec MH reform. While local MH steering committees had previously existed, in all networks, each HSSC was required to produce a clinical MH project or strategic plan for network service delivery in conjunction with their respective MH service providers, under the terms of the reform. The clinical projects for the WH- and LN-Groups established working committees charged with identifying gaps in MH services and possible remedies. Steering committees for the PH- and SN-Groups reportedly lost their decision-making powers after disseminating their clinical projects. Overall, participation in the local steering committees of regional organizations such as PHs and some community organizations became more difficult under the reform. Performance indicators introduced into MH planning were not sufficiently utilized, and they failed to capture overall quality issues and territorial realities adequately.

Shared-cared, favouring contacts between specialized services and primary care was introduced through the respondent-psychiatrist role. Other objectives focused on consolidating expertise within the HSSC-MH teams, and increasing services. As shared-care began in the PH-Group, a culture clash ensued between respondent-psychiatrists and the HSSC-MH teams. Psychiatrists did not have a good perception of their role, and clarification became necessary. In the WH-Group, shared-care existed in one of the two territories, where psychiatrists were deployed from a general hospital. Shared-care started later in the SN-Group, as respondent-psychiatrists were reportedly uncomfortable in this role. They resisted involvement in the clinical decisions of GPs, and declined to take responsibility for referrals to specialized MH services. In the LN-Group, shared-care was successfully implemented in most territories.

## Barriers and facilitators to network integration

Underestimating the importance of operational mechanisms such as clinical evaluation tools and best-practice guidelines in implementing the MH reform was reportedly the main barrier to network integration. Other factors hindering the network integration effort included the persistence of a strong hospital-centrism in some networks, resistance to change, and fears among some organizations of losing their autonomy. Furthermore, specific barriers concerning the integration of respondent-psychiatrists were reported, such as poorly defined roles, negative perceptions among GPs regarding the usefulness of respondent-psychiatrists, and absence of financial incentives for GPs. A strong facilitator to network integration, according to participants, was the action of the MH-NCE, followed by the work of liaison officers, organizational leadership and the existence of patient-centred and needs-based philosophies shared by most services providers.

## Discussion

At least in theory, the Quebec MH Action Plan included most of the essential components for successful implementation of the reform and for improved network integration: the focus on development of primary care services and MH teams, on patient needs and better access to services, on quality and continuity of care, on strong leadership, etc. [26,38]. However, the results of this study demonstrate that the objectives of the reform were not fully met, and that the implementation of integrated networks was not uniformly completed across territories. Although local networks were in a process of development and expansion [39] and basic collaboration existed within all the groups, none of them demonstrated all the characteristics of a fully integrated network [24].

The objectives of the MH Action Plan were best achieved in the LN-Group, followed by the PH-Group. Most implementation barriers involved organizational characteristics as hypothesized; they included frequent staff turnover, resistance to change, leadership problems, lack of inter-organizational collaboration. These factors are usually underlined in the literature as important barriers to integration [23,37,38,40]. Implementation context, reform characteristics, and restricted use of integration strategies also hampered implementation of the MH reform in all networks.

The LN-Group met the recommended wait times for evaluation and treatment in primary care and in specialized MH services more closely than other groups, and hired more psychiatrists in primary care teams, which allowed for more intensive care and care pathways. Conditions in this group were more favourable at onset of the MH reform. Territories in the LN-Group also implemented several recommendations advanced by the MH reform, but had already established collaboration between the HSSC and network service providers well before introduction of the Plan. This collaboration continued after development of their clinical project. Thus, the MH reform did not break with the established vision and practices for the LN-Group, but reinforced continuity. Moreover, the LN-Group had relatively fewer clients with severe MHDs and co-occurring MHD-SUDs in their primary care services. Leadership from the HSSC in the LN-Group was also fully integrated, with critical support from the Regional Agency including financing for ACT and ICM services that were costly to implement, but more cost-effective than traditional services in the long term [41]. Sites with strong leadership are more likely to uphold fidelity standards in implementing innovations [10,42,43]. Furthermore, it is important to note that network leadership was not necessarily assumed by one manager alone, but may have been a collective effort [39].

By contrast, networks in the SN-Group experienced adverse conditions, including poverty, many severe MHD cases in primary care, and significant staff turnover, which may explain their failure to achieve targeted wait times for service, and to adopt the proposed integration strategies, as well as their need to solicit assistance from the MH-NCE. Despite difficult conditions, the SN-Group reported satisfying interactions among primary care teams and MH specialized services, medical clinics and community organizations. The WH-Group also confronted difficult conditions due to the lack of specialized MS services in their territories, underfunding and a high prevalence of primary care clients with co-occurring MH-SUDs. The relatively high implementation of integration strategies in the WH-Group seemingly aimed to remedy the difficulties. Smaller organizations with limited resources in the SN- and WH-Groups also experienced difficulties with staff turnover [6]. The ability to retain qualified staff is a key element for successful implementation of reforms and maintenance of service quality, yet acquiring new staff may also imply greater openness to innovation [44]. Rivalry between the HSSC and the PH in the PH-Group greatly hindered MH reform; whereas the dispersion of power among organizations facilitated networking. Resource abundance, and scarcity, may also have worked against both network integration and reform implementation [45]. However, an abundance of MH services allowed the PH-Group to introduce the greatest variety of integration strategies, including standardized evaluation/clinical tools, respondent-psychiatrists, service agreements, liaison officers, joint training and shared-staff, all of which favoured reform and suggested a trend in service-rich networks that is confirmed by the literature [45].

Resistance to change was identified as an important barrier to MH reform [42,43], emanating from ideological conflict [6] and occurring in this study among psychiatrists in the SN-Group who adopted a hospital-centred model. In another case, the Quebec Psychiatric Association boycotted the uptake of shared-care until 2010. Given that psychiatrists and GPs were so central to MH service delivery [38], their difficulties and resistance seriously hampered the reform. Resistance to change may also have resulted from the simultaneous implementation of overly complex and multiple changes [6]. Those already grappling with global reform of the Quebec Health and Social services system (Law 83) experienced the MH reform as an additional burden. When implementation timelines are too compressed, adequate communication with clinicians and organizations may not occur [46]. Sufficient time is required for stakeholders to integrate and experiment with new services and practices [47], as well as new information, support and training [42].

Implementation difficulties were also strongly related to characteristics of the MH reform itself, in simultaneously introducing several new structures and services instead of facilitating reform through a sequential implementation process [6]. Moreover, there was little focus in the MH reform on operational mechanisms to support the newly proposed structures or services, such as integration strategies, which may explain the difficulties encountered by the WH- and SN-Groups in operating the one-stop service or HSSC-MH primary care teams.

The implementation of several administrative (functional) and clinical integration strategies is essential for successful network integration [48]. However, results indicate that few integration strategies were strongly employed among the different groups. The main integration strategies adopted involved the administrative level, which may explain the high level of satisfaction among MH primary care teams with other services or organisations. Administrative (or functional) integration strategies facilitate collaboration between distinct levels of care (primary care, specialized services) and organisations [22], and, in turn, increase access to care, and care continuity. However, the main administrative strategies adopted, such as referral procedures, required little organizational involvement [49]. According to the literature, networks that develop more formalized mechanisms, such as service agreements and liaison officers, produce better integration [14,50,51]. By contrast, clinical strategies (clinical/evaluation tools and clinical approaches) were weakly implemented by most groups. The literature underscores the importance of clinical integration as central to the service integration process [52,53]. The fact that the Quebec HM reform did not provide sufficient guidance on clinical processes may explain why clinical strategies were underutilized. In some countries like England [54] and Australia [55], guidelines on clinical evaluation tools, best practices, performance assessment frameworks and specific indicators for system evaluation were provided at the outset of their MH reforms [16].

The more successful utilization of integration strategies by the PH-and WH-Groups was probably due to a greater willingness to solicit assistance from the MH-NCE. The presence of former PH clinicians in primary care teams within the PH-Group may explain why standardized evaluation tools were also more fully utilized. Furthermore, while training or sustained supervision were not prioritized by the MH reform, research suggests that the implementation of evidence-best practices requires sophisticated skills that can only be acquired through a training infrastructure that includes both theoretical and practical approaches [56]. Finally, the MH reform fell short in terms of the need to strengthen the implementation of performance indicators. A rigorous evaluation of practice quality and fidelity is a major determinant of success according to implementation science sources [57].

## Limitations

This study has a number of limitations. First, the results may not be generalizable to other countries with divergent healthcare systems. By the same token, it may be difficult to generalize the results across Quebec, despite efforts to provide a representative sample of networks. Second, study participants may have over- or under-estimated the actual degree of reform implementation or integration in their networks. In order to neutralize participant bias, results were compared and validated using a mixed-method approach and data triangulation, and the collaboration of a research advisory committee was sought. Third, since our study was cross-sectional, the perception of stakeholders may have changed over time. Finally, the viewpoints expressed by participants tended to be more convergent than divergent.

## Conclusions

This is the first study to evaluate implementation of the Quebec MH reform, based on findings from four network groups, and has potentially important implications for international MH reform. Results show that implementation of the MH reform was not fully achieved in any of the territorial groups, although implementation was more advanced among groups that enjoyed more favourable conditions from the outset. Organizational factors were the strongest barriers to implementation in most networks. We propose the following seven recommendations for eliminating barriers to successful reform implementation and network integration as suggested by the findings, with potential relevance for MH reform elsewhere: First, decision makers should ensure that each network has the necessary material and human resources to promote staff retention and adequately meet their population needs. Second, successful implementation takes time, and new measures must be implemented sequentially. Third, since joint training facilitates the creation of common values and practices among professionals and within organizations, a systematic training program on screening and assessment tools as well as clinical approaches for MHD should be developed at the provincial level (or national level in other countries). Fourth, support from governmental agencies or sustained supervision from other resources should be systematically enlisted by all networks implementing evidence-based best practices. Fifth, performance indicators that better reflect quality issues and territorial realities should be established. Six, measurement tools evaluating the process and the impact of integration should be used systematically. Finally, the improvement of integrated networks and a better continuum of care for clients with MHDs depend crucially on the implementation of more formalized integration strategies.
